# The endoplasmic reticulum mitochondrial calcium cross talk is downregulated in malignant pleural mesothelioma cells and plays a critical role in apoptosis inhibition

**DOI:** 10.18632/oncotarget.4370

**Published:** 2015-06-19

**Authors:** Simone Patergnani, Carlotta Giorgi, Stefania Maniero, Sonia Missiroli, Pio Maniscalco, Ilaria Bononi, Fernanda Martini, Giorgio Cavallesco, Mauro Tognon, Paolo Pinton

**Affiliations:** ^1^ Department of Morphology, Surgery and Experimental Medicine, Section of Pathology, Oncology and Experimental Biology, Laboratory for Technologies of Advanced Therapies (LTTA), University of Ferrara, Ferrara, Italy; ^2^ Department of Morphology, Surgery and Experimental Medicine, Section of General and Thoracic Surgery, University of Ferrara, Ferrara, Italy

**Keywords:** calcium, mesothelioma, mitochondria, apoptosis, therapy

## Abstract

The failure of apoptosis may contribute to the formation of cancer and to its resistance to therapy. Malignant pleural mesothelioma (MPM) is an aggressive tumor that responds poorly to standard chemo- and radio-therapies. Several studies have demonstrated that a plethora of oncogenes and tumor suppressors contribute to MPM onset/progression. Importantly, most of these genes are involved in the regulation of calcium (Ca^2+^)-handling. Cellular Ca^2+^ signaling is an important regulator of many physiological processes, and it has been widely reported to participate in the regulation of apoptotic cell death in cancer cells and tissues. However, in MPM the role of cellular Ca^2+^ has been poorly investigated. Therefore, we examined whether Ca^2+^ is involved in MPM. We found that mesothelioma cell lines and short-term cultures obtained from MPM-affected patients exhibited a critical dysregulation in Ca^2+^ signaling. We determined that this characteristic was associated with resistance to apoptotic stimuli and that correction of intracellular Ca^2+^ signaling resulted in the rescue of efficient apoptotic responses. In addition, we discovered that mitochondrial Ca^2+^-uptake plays a pivotal role as an inducer of apoptosis in MPM. Altogether, these findings suggest the identification of new MPM markers, which in turn could be potential targets for new therapeutic approaches.

## INTRODUCTION

Human malignant pleural mesothelioma (MPM) is a highly aggressive neoplasm that leads to patient death within a few months or years [1–3]. Presently, no standard curative therapy exists for MPM, and there are no recognized specific markers for the diagnosis of MPM. In addition, little is known regarding the apparent lack of apoptosis in mesothelioma cells [3–5].

Apoptosis is a highly tuned mechanism that is regulated by distinct and complex pathways. This process can be induced by a variety of physiological and pharmacological stimuli [6], including changes in the Ca^2+^ distribution within intracellular compartments [7]. Specifically, evidence indicates that both Ca^2+^ release from intracellular pools and capacitative Ca^2+^ influx through Ca^2+^ release-activated Ca^2+^ channels are sufficient to trigger apoptosis [8–12]. Additionally, mitochondria play a critical role in Ca^2+^ homeostasis and apoptosis [13, 14]. In conjunction with incremental changes in the cytosolic Ca^2+^ levels, similar or even larger levels of mitochondrial Ca^2+^ ([Ca^2+^]_m_) uptake have been observed [15]. [Ca^2+^]_m_ overload is a pro-apoptotic mechanism that induces mitochondrial swelling, which perturbs or ruptures the outer mitochondrial membrane, inducing the release of mitochondrial apoptotic factors into the cytosol [16, 17].

A large body of evidence has indicated the potential role of Ca^2+^ in the onset or progression of cancer, highlighting Ca^2+^ handling as a potential target for the pharmacological treatment of tumors [18]. In particular, perturbations in Ca^2+^ homeostasis and alterations in the principal pumps and channels responsible for appropriate Ca^2+^ handling have been found in cancers of different histotypes [19–23]. It has been reported that the mineral asbestos, which is considered the main cause of MPM onset/progression, induces an endoplasmic reticulum (ER)-stress response, consequently altering ER-Ca^2+^ ([Ca^2+^]_ER_) release [24]. Furthermore, several compounds considered to be promising molecules for the treatment of MPM are capable of inducing oxidative stress, leading to ER-stress and Ca^2+^ release [24]. Moreover, mutations and alterations in the expression of many oncogenes and tumor suppressor genes, which are involved in the control of both apoptosis and Ca^2+^ handling, have been identified in mesothelioma specimens and are considered responsible for MPM onset/progression [25–28].

Collectively, these data suggest the importance of crosstalk between the dysregulation of Ca^2+^ homeostasis, apoptosis and cancer progression. It is noteworthy that the relevance of these relationships to human MPM could be highly significant for the development of new therapeutic approaches for this cancer, which at present is neglected.

In this study, MPM-specimens exhibited a global reduction in Ca^2+^ physiology. This characteristic involved the inhibition of Ca^2+^ transfer from the ER to the mitochondria, preserved mitochondrial integrity and protected against Ca^2+^-mediated apoptosis. Furthermore, for the first time, we demonstrated that various approaches promoting intracellular Ca^2+^ accumulation restored the typically deficient levels of apoptosis in MPM cells back to physiological levels.

## RESULTS

### MPM specimens exhibit dysregulated cellular Ca^2+^ signaling

**Figure 1:**
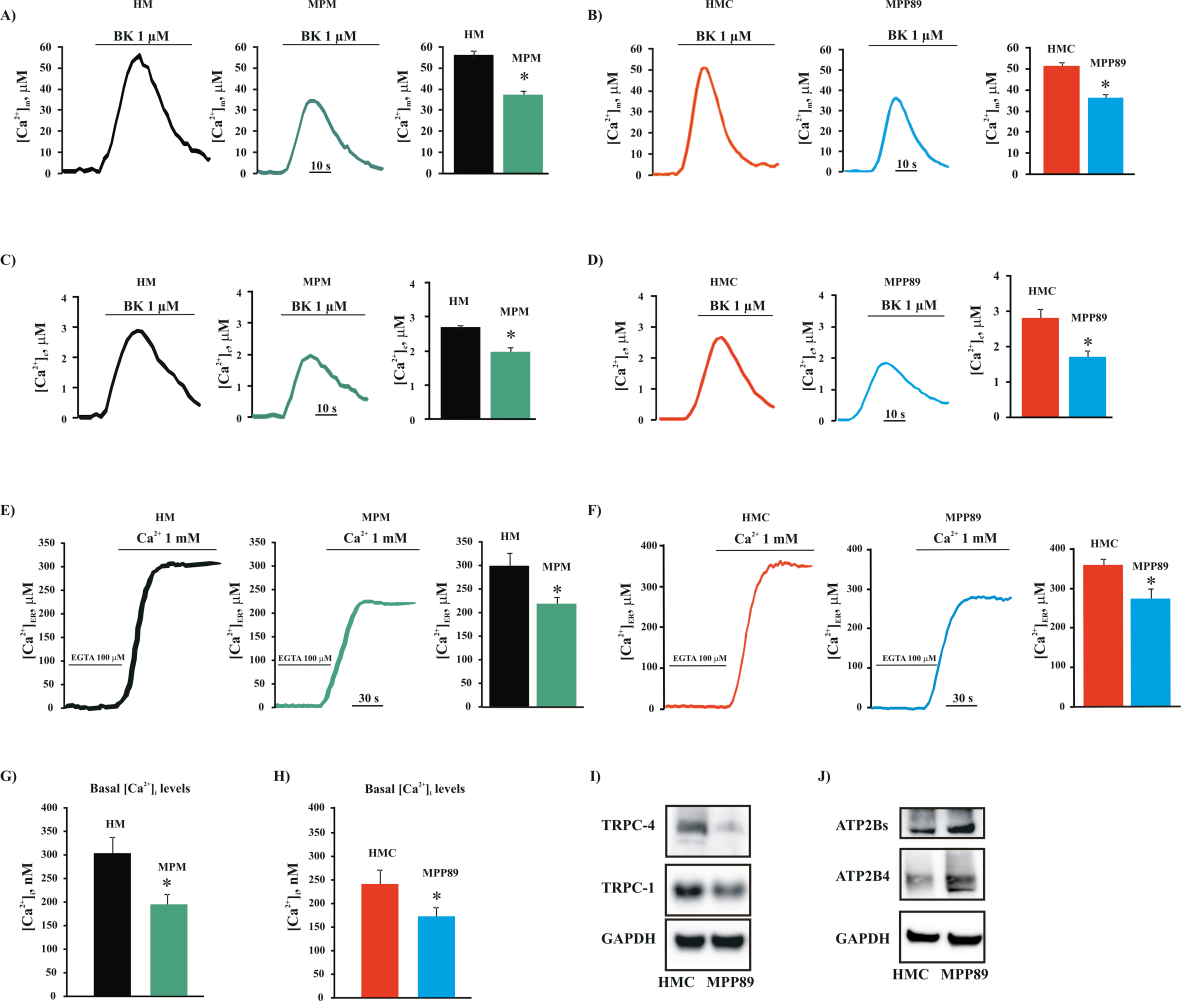
Intracellular and intraorganellar Ca^2+^ homeostasis in MPM cells Representative mitochondrial **A.** and cytosolic **B.** Ca^2+^ measurements in primary cell cultures obtained from healthy (HM) and MPM-affected patients (MPM) (peak amplitude [Ca^2+^]_m_: 56.83 ± 1.32 μM [HM], 37.89 ± 1.44 μM [MPM], *n* = 16; peak amplitude [Ca^2+^]_c_: 2.87 ± 0.43 μM [HM] vs. 2.02 ± 0.34 μM [MPM]; *n* = 18). Likewise, the alteration of mitochondrial **C.** and cytosolic **D.** Ca^2+^ handling was assessed in normal mesothelial (HMC) and malignant mesothelioma (MPP89) cell lines (peak amplitude [Ca^2+^]_m_: 51.36 ± 1.87 μM [HMC], 36.81 ± 1.98 μM [MPP89], *n* = 32; peak amplitude [Ca^2+^]_c_: 2.83 ± 0.34 μM [HMC], 1.75 ± 0.23 μM [MPP89], *n* = 37). Additionally, the steady-state [Ca^2+^]_ER_ was analyzed in primary cell cultures obtained from healthy (HM) and MPM-affected patients (MPM) **E.** and in normal (HMC) and MPM (MPP89) **F.** commercial cell lines (steady state [Ca^2+^]_ER_: 217.86 ± 14.34 μM [MPM], 298.45 ± 22.21 μM [HM], *n* = 12; 283.67 ± 18.11 μM [MPP89], 364.49 ± 11.81 μM [HMC], *n* = 14). Representative traces are shown. Next, primary cell cultures **G.** and commercial cell lines **H.** were loaded with the Ca^2+^-indicator FURA-2/AM to analyze the basal [Ca^2+^]_i_ (basal [Ca^2+^]_i_ in commercial cell lines: 238.73 ± 18.24 nM [HMC], 174.78 ± 11.53 nM [MPP89], *n* = 16; basal [Ca^2+^]_i_ in primary cell cultures: 304.48 ± 31.65 nM [HM], 193.98 ± 22.72 nM [MPM], *n* = 14). Finally, the protein expression of C-type TRPCs **I.** and ATP2Bs **J.** in normal and mesothelioma cell lines was assessed by immunoblotting. Membrane protein samples (15 μg/lane) were loaded and probed using specific antibodies. GAPDH was used as a loading control. All graphs display the means ± SEM. *p < 0.01. Abbreviations: BK, bradykinin; KRB: Krebs ringer buffer.

Our study aimed to investigate whether intracellular Ca^2+^ plays a key role in the progression of MPM. To measure intracellular Ca^2+^ homeostasis, we used aequorin probes targeted to different subcellular compartments. These experiments employed the inositol triphosphate agonist bradykinin (BK), which is known to act via distinct surface membrane receptors to promote ER-Ca^2+^ mobilization [29]. The data shown in Figure 1A demonstrate that short-term cell cultures derived from biopsies of MPM-affected patients (MPM) exhibited a marked reduction in [Ca^2+^]_m_ uptake in response to stimulation of the cells with BK when compared with cell cultures derived from biopsies of healthy individuals (HM). In parallel, we found the same features in MPM commercial cell lines (Figure 1B and Supplementary Figure S1A–1B).

To investigate the possibility that this reduced Ca^2+^ signaling was not restricted to the mitochondrial compartment, we monitored the Ca^2+^ concentrations in the cytosol ([Ca^2+^]_c_). In MPM cells, the [Ca^2+^]_c_ increases were significantly smaller than those in control cells (Figure 1C–1D). Given that the concentrations of Ca^2+^ in the mitochondria and cytosol are highly dependent on the amount of Ca^2+^ in the ER, we investigated the Ca^2+^ concentrations in the ER compartment [Ca^2+^]_ER_. We found that the steady state [Ca^2+^]_ER_ in the mesothelioma cell was markedly lower than in HMC controls (Figure 1E–1F).

The ER constitutes the principal Ca^2+^ store and participates in the initial rapid increase in [Ca^2+^]_c_ by supplying Ca^2+^ via the inositol 1,4,5-trisphosphate receptors (ITPRs). The ER also participates in the subsequent decrease in [Ca^2+^]_c_ by removing Ca^2+^ from the cytoplasm and recovering the internal Ca^2+^ stores through the action of sarco- and endoplasmic reticulum Ca^2+^-ATPases (ATP2A2). It is clear that ATP2A2 pumps are the principal regulator for the maintenance of [Ca^2+^]_ER_. One of the most common compounds used to induce intracellular Ca^2+^ accumulation, the sesquiterpene thapsigargin (TG), is a specific and potent inhibitor of ATP2A2. Taking advantage of this feature, we decided to evaluate the native store filling of the ER compartment in normal and mesothelioma cells. Cells were loaded in Ca^2+^-free medium with the Ca^2+^-indicator Fura-2-acetoxymethylester (FURA-2/AM) for 30 min, and the levels of the thapsigargin-releasable Ca^2+^ were assessed. We found that in MPM cells, the thapsigargin-dependent intracellular Ca^2+^ elevation was significantly lower when compared with those observed in HMC cells (Supplementary Figure S1C–1D). These results suggest that the native store filling of the ER may be seriously affected. As yet reported, the amplitude of the ER-Ca^2+^ transient is regulated by different ER-resident protein, such as ATP2A2 and ITPR3. For these reasons, we investigate whether the alteration in Ca^2+^ handling could be due to a change in the expression of an ER-Ca^2+^ binding proteins.

Interestingly, we found that in MPM cells, the levels of the ATP2A2 and ITPR3 proteins were seriously affected. By contrast, we did not observe alterations in the expression of other ER-Ca^2+^ binding proteins, such as CALNX (Supplementary Figure S1E). Overall, these data may suggest that MPM cells display a critical perturbation in [Ca^2+^]_ER_, most likely due to a lack in the activity of two of the main proteins involved in Ca^2+^ release and Ca^2+^ reuptake at ER level.

It is well established that the triggering of apoptosis involves Ca^2+^ influx via mitochondrial, cytoplasmic and ER-mediated mechanisms [30]. Similarly, it is well known that transformed cells lacking apoptosis exhibit a reduced capacity to maintain an excessive intracellular Ca^2+^ concentration ([Ca^2+^]_i_); this is often due to store-operated Ca^2+^ entry (SOCE) that is no longer functional and/or suppressed by Ca^2+^ efflux across the plasma membrane (PM) [8]. To investigate whether this remodeling of Ca^2+^ signaling is also a characteristic of MPM, a cytosolic aequorin Ca^2+^ probe and the Ca^2+^ indicator FURA-2/AM were used to measure capacitative and basal Ca^2+^ influx, respectively. The data reported in Supplementary Figure S1F show that the MPM cell lines exhibited reduced Ca^2+^ influx activity. Indeed, MPP89 cells displayed a significant reduction in their ability to take up Ca^2+^ into the cytosolic compartment compared with HMC. In accordance with these data, the basal [Ca^2+^]_i_ was markedly lower in the MPM cells compared with the control cells (Figure 1G–1H).

Many studies have established that multiple cancer types are associated with alterations in the expression of proteins involved in the movement of Ca^2+^ across the PM, specifically Ca^2+^ channels and pumps such as TRP-channels (TRPCs) and PM Ca^2+^-ATPase pumps (ATP2Bs) [8].

Thus, we assessed the expression of TRPCs and ATP2Bs in MPM and HMC cells. We found that the protein levels of the TRPC isoforms TRPC1 and TRP4C3 were substantially reduced in mesothelioma cells compared with HMC cells (Figure 1I). Furthermore, consistent with the basal intracellular Ca^2+^ measurements reported in Figure 1G–1H, we found that expression of the ATP2Bs, including isoform ATP2B4, was increased in MPM cells (Figure 1J). Importantly, it has been demonstrated that this ATP2B4 isoform is strongly involved in tumorigenesis [31].

### MPM specimens exhibited alterations of the apoptotic machinery

**Figure 2:**
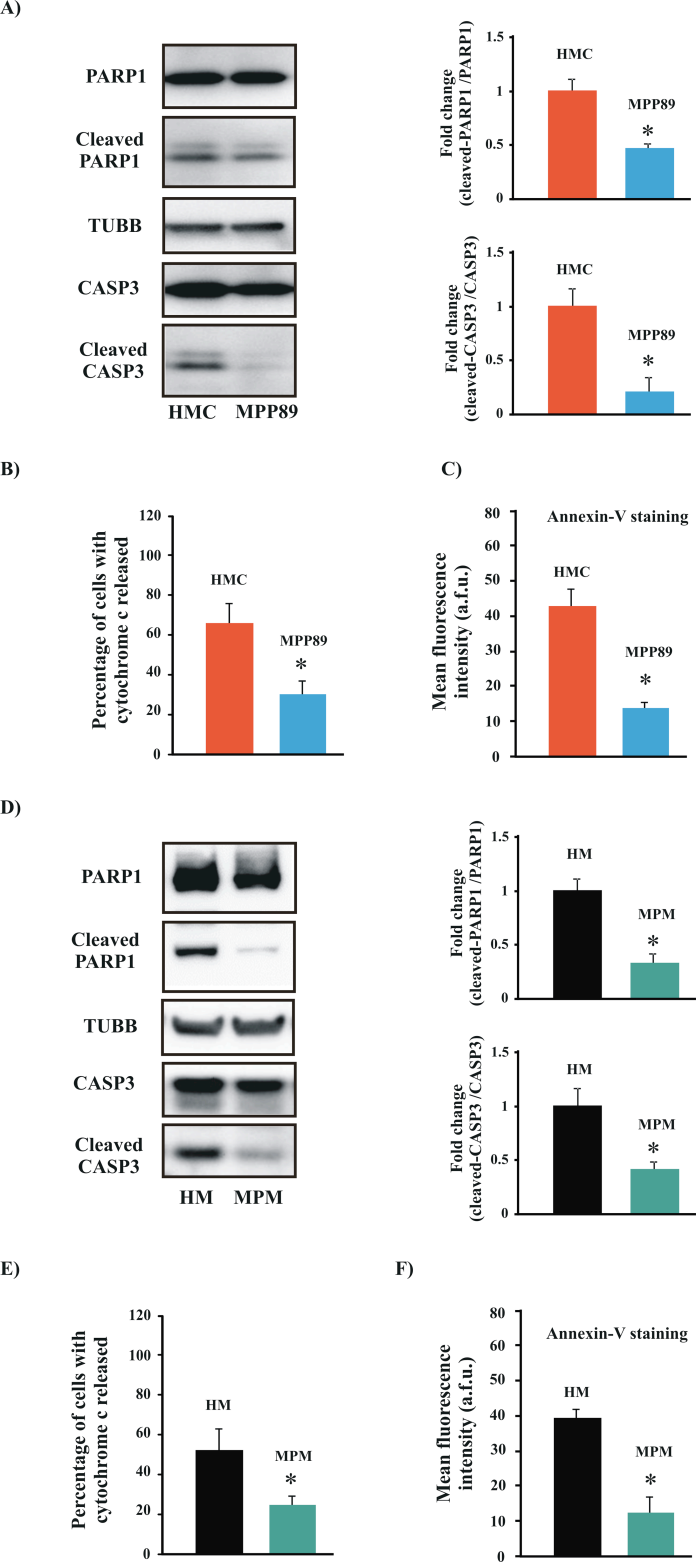
Measurements of apoptotic levels in MPM samples **A.** Representative immunoblot showing cleavage of the apoptotic markers PARP1 and CASP3 in normal mesothelial (HMC) and mesothelioma (MPP89) cell lines. The upper and lower graphs are expressed as the means ± SEM and depict the fold-changes in the cleaved-CASP3/CASP3 ratio and the cleaved-PARP1/PARP1 ratio, respectively. **B–C.** Analysis of cytochrome c release and Annexin-V staining. The subcellular distribution of cytochrome C (B) was visualized using an anti-cytochrome c antibody (percentage of cells with cytochrome c released: 62.76 ± 15.42% [HMC] vs. 29.32 ± 7.56% [MPP89]; *n* = 7). For detection of PS on the extracellular surface of the PM, cells were stained with Annexin-V (Annexin-V fluorescence intensity: (41.21 ± 7.32 a.f.u. [HMC] vs. 13.45 ± 2.98 a.f.u. [MPP89]; *n* = 8) (C) The same experiments were replicated in short-term cell cultures obtained from healthy (HM) and MPM-affected patients (MPM). As reported in the text, the apoptotic machinery in the primary MPM cells was affected **D–F.** (percentage of cells with cytochrome c released: 56.87 ± 11.23% [HM] vs. 22.91 ± 3.65% [MPM]; *n* = 5; (Annexin-V fluorescence intensity: 38.89 ± 5.01 a.f.u. [HM] vs. 11.77 ± 4.17 a.f.u. [MPP89]; *n* = 5). All graphs display the means ± SEM. *p < 0.01.

As described above, Ca^2+^ plays various key physiological roles in normal human cells. Importantly, Ca^2+^ overload has been considered to be a final common pathway of apoptosis. Because we found significant differences in Ca^2+^ homeostasis between MPM and HMC cells, we sought to examine the rate of apoptosis under our experimental conditions. We found that HMC cells display significantly higher apoptotic levels than those observed in MPM cells (Figure 2A–2C and Supplementary Figure S2). Furthermore, these findings were clearly confirmed by analyzing the levels of apoptosis in other mesothelioma specimens (Supplementary Figure S2).

Notably, a major criticism of cell lines is that they grow indefinitely in the laboratory and could differ genetically from primary tissue, accumulating new mutations as they adapt to their artificial environment. To address this issue, we performed the same apoptosis assays on primary cell cultures obtained from biopsies of healthy individuals (HM) or MPM-affected patients. The results of these studies confirmed our hypothesis. Indeed, mesothelioma specimens exhibited a sustained downregulation of the apoptosis pathway compared with normal mesothelial cells (Figure 2D–2F).

### Dysregulated cellular Ca^2+^ signaling exerts adverse effects on the execution of the mitochondrial apoptotic process and on the sensitivity to apoptotic stimuli

Due to the large differences in the modulation of Ca^2+^ homeostasis and the activity of the apoptotic machinery between normal mesothelial and mesothelioma cells, we sought to examine whether these two aspects of cell death were interrelated. Ca^2+^ transfer from the ER to the mitochondria has been shown to be involved in several models of apoptosis. Importantly, it has been well demonstrated that this event is directly responsible for mitochondrial Ca^2+^ overload, mitochondrial permeability transition (MPT) pore opening and caspase-mediated cell death [6]. Various apoptotic stimuli, such as menadione, H_2_O_2_ and C_2_-ceramide (C_2_), induce the movement of Ca^2+^ from the ER to the mitochondrial compartment [32, 33]. Based on these findings, we investigated whether treatment with these stimuli promoted the apoptotic process via the use of Ca^2+^ as a cofactor that sensitizes mesothelioma cells for apoptotic cell death.

**Figure 3:**
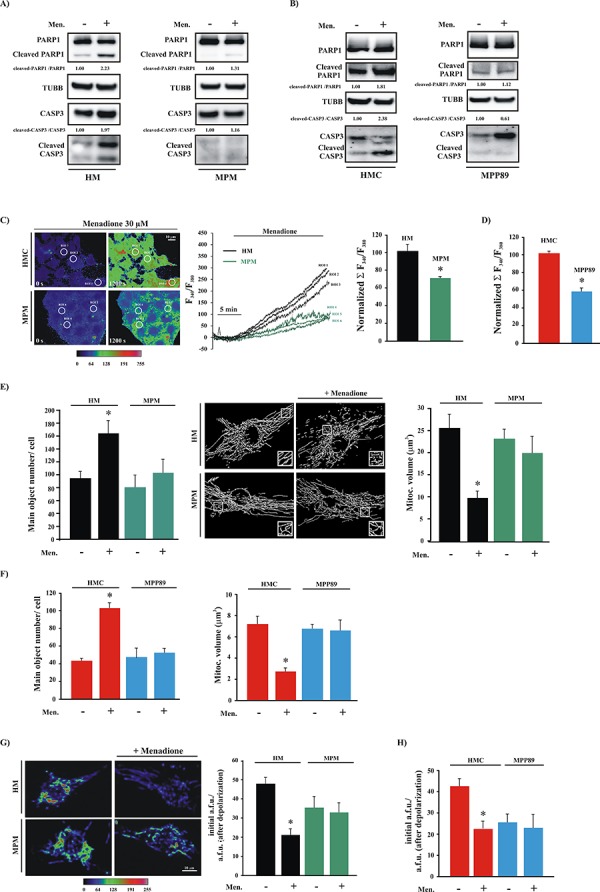
MPM cells are insensitive to pro-apoptotic stimuli **A.** Primary cell cultures (HM and MPM) and **B.** cell lines (HMC and MPP89) cells were treated with 30 μM menadione (Men) or left untreated. After 30 min, the cells were subjected to immunoblotting for the indicated proteins. Normalized fold-change values in the cleaved-CASP3/CASP3 ratio and cleaved-PARP1/PARP1 ratio are shown. **C.** Cells were loaded with FURA-2/AM and stimulated with 30 μM Menadione (Men) for 30 min. The kinetic properties of the Ca^2+^ response are presented as the ratio of fluorescence at 340 nm/380 nm. Representative images captured at different time points (0 s and 1200 s) are shown in the left panel and are displayed in pseudo-color. The middle panel depicts the representative kinetic properties of specific ROIs (regions of interest) in the left panel (black for HM and green for MPM). **D.** Normalized fluorescence ratio 340/380 nm of the Ca^2+^ response after stimulation with 30 μM menadione in HMC and MPP89 cells. Representative images and kinetics are shown in Supplementary Figure S3D **E.** Analysis of mitochondrial structures in HM and MPM cells transfected with a green fluorescent protein targeted to the mitochondria (mtGFP). The acquired images were deconvoluted, reconstructed and quantitatively analyzed. Representative reconstructed 3D images are shown. The data represent the means ± SEM of the mitochondrial number/cell (left) and the volume of an individual mitochondrion (right) (number of mitochondria: 94.67 ± 8.73 [HM], 164.43 ± 16.35 [HM+Men], *n* = 12; 81.03 ± 17.77 [MPM], 102.32 ± 21.37 [MPM+Men], *n* = 11); (volume of individual mitochondria: 25.98 ± 3.79 μm^3^ [HM], 9.31 ± 2.19 μm^3^ [HM+Men], *n* = 12; 23.71 ± 1.98 μm^3^ [MPM] vs. 19.89 ± 4.45 μm^3^ [MPM+Men]; *n* = 11). **F.** Quantitative analysis of mitochondrial parameters in normal (HMC) and mesothelioma (MPP89) cell lines: number of mitochondria per cells (41.23 ± 2.31 [HMC], 101.29 ± 5.87 [HMC+Men], *n* = 21; 43.11 ± 8.38 [MPP89], 49.67 ± 4.33 [MPP89+Men], *n* = 19) and volume of individual mitochondria: 7.43 ± 1.11 μm^3^ [HMC], 2.67 ± 0.63 μm^3^ [HMC+Men], *n* = 21; 6.88 ± 0.45 μm^3^ [MPP89] vs. 6.08 ± 1.54 μm^3^ [MPP89+Men]; *n* = 19). Representative images are shown in Supplementary Figure S3E. **G.** Cultured cells were loaded with TMRM and imaged via confocal microscopy to determine the Ψm. Representative images and quantitative results (means ± SEM) are shown (TMRM intensity a.f.u.: 48.22 ± 3.52 [HM], 21.76 ± 2.88 [HM+Men]; *n* = 13; *p* < 0.01; 35.11 ± 7.31 [MPM] vs. 31.22 ± 5.99 [MPM+Men]; *n* = 12). **H.** Bar graph showing quantification of the TMRM signals measured by fluorescence microscopy in HMC and MPP89 cells. (TMRM intensity a.f.u.: 41.86 ± 3.10 [HMC], 20.99 ± 4.77 [HMC+Men]; *n* = 12; *p* < 0.01; 24.09 ± 5.11 [MPP89] vs. 21.11 ± 6.54 [MPP89+Men]; *n* = 19). To establish the mitochondrial Ψm levels, the ratio between the initial TMRM fluorescence (initial a.f.u.) and the residual TMRM fluorescence (obtained after FCCP addition, a.f.u. after depolarization) was calculated. The values obtained represent the amount of the potential-indicating probe incorporated in the different experimental conditions. Representative images are shown in Supplementary Figure S3F. *p < 0.01.

First, we analyzed the biochemical characteristics of the apoptotic process in cells subjected to oxidative stress (menadione). As expected, we found that apoptotic activity was strongly induced in HMCs treated with menadione, but not in MPM cells, where the ability of the oxidative agent to induce apoptosis was abrogated (Figure 3A–3B and Supplementary Figure S3A–3B). Then, we measured apoptosis after the administration of other apoptotic agents. We found that treatment with H_2_O_2_ and C_2_ did not activate the apoptotic program in mesothelioma cells (Supplementary Figure S3C).

As mentioned above, exposure to different apoptotic stimuli may trigger or enhance the amount of Ca^2+^ released from the ER. Thus, we investigated whether the apoptotic characteristics of menadione are associated with Ca^2+^ signaling via the ER. MPM and HMC cells were loaded with the Ca^2+^ indicator FURA-2/AM and monitored during stimulation with menadione. The addition of menadione to HMCs caused a rapid elevation of FURA-2-associated intracellular fluorescence during the following 30 min. In contrast, this increase was significantly reduced in MPM cells (Figure 3C–3D and Supplementary Figure S3D).

During apoptosis, mitochondria undergo massive fragmentation with concomitant permeabilization of the outer mitochondrial membrane and cytochrome c release. Furthermore, the mitochondrial membrane potential (Ψ_m_) collapses, and the amount of ROS produced is augmented [34]. As demonstrated, we found remarkable morphological injury to the mitochondria, prominent dissipation of the Ψ_m_ and a notable augmentation of mitochondrial ROS production in HMC cells. No difference was detected in MPM cells treated with menadione (Figure 3E–3H and Supplementary Figure S3E–3H).

**Figure 4:**
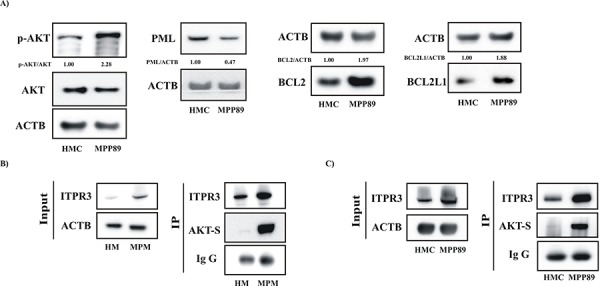
Apoptosis-related proteins regulating the core mechanism of the Ca^2+^-dependent apoptotic pathway are involved in MPM **A.** Protein expression levels of the primary cofactors involved in the regulation of Ca^2+^ handling. Lysates prepared from HMC and MPP89 cells were loaded on a precast gel and transferred to nitrocellulose membranes. These membranes were probed for the desired proteins using specific primary antibodies. Normalized fold-change values in the ratio of p-AKT/AKT, PML/ACTB, BCL2/ACTB and BCL2L1/ACTB are shown. **B–C.** The AKT-mediated ITPR3 phosphorylation was investigated in primary cells obtained from healthy and MPM-affected individuals (B) and in commercial cell lines (C) Cell lysates were immunoprecipitated with ITPR3 antibody. The immunoprecipitates were immunoblotted with AKT-S antibody or ITPR3 antibody.

In recent years, important studies have indicated a key role for Ca^2+^ in apoptotic cell death based on the finding that specific oncogenes and tumor suppressor genes regulate cell death by perturbing intracellular Ca^2+^ homeostasis [35]. We therefore examined whether mesothelioma cells displayed some of these characteristics. We detected a prominent increase in the protein expression of activated AKT, BCL2 and BCL2L1 in MPM cells compared with normal mesothelial cells (Figure 4A). Importantly, we also found a significant reduction in the protein levels of the oncosuppressor PML (Figure 4A). Altogether, these findings may explain the observed dysregulated cellular Ca^2+^ signaling. Several works highlight the presence of a direct interaction between PML and AKT to modulate the Ca^2+^ handling. Furthermore, it has been widely reported that IP3 receptors (ITPRs) are substrates for AKT [36] and, in particular, that AKT controls the Ca^2+^-dependent apoptotic process by regulating ITPR3 activity [37]. Thus, we investigated the possible relationship between ITPR3 and AKT in mesothelioma cells. MPM cells (primary and commercial cell lines) were collected, lysed and immunoprecipitated with ITPR3 antibody.

Next, immunoprecipitated fractions were assayed for phosphorylation using an AKT-substrate (AKT-S) antibody. The data reported in Figure 4B–4C show that the endogenous type III ITPR was highly phosphorylated by AKT in mesothelioma cells.

### The recovery of Ca^2+^ homeostasis enhances [Ca^2+^]_m_ uptake, thereby promoting apoptotic activity

To evaluate if the disruption of Ca^2+^ homeostasis is a major contributor to the absence of the apoptotic process that is typical of MPM, we attempted to force Ca^2+^-influx into mesothelioma cells because higher [Ca^2+^]_i_ should increase mitochondrial Ca^2+^ uptake, thereby activating the apoptotic machinery. MPP89 cells were pretreated with a high external [Ca^2+^] (10 mM) or with sodium orthovanadate (Na_3_VO_4_, NaV, 1 μM), a general inhibitor of P-type ATPases, in particular ATP2Bs [38].

**Figure 5:**
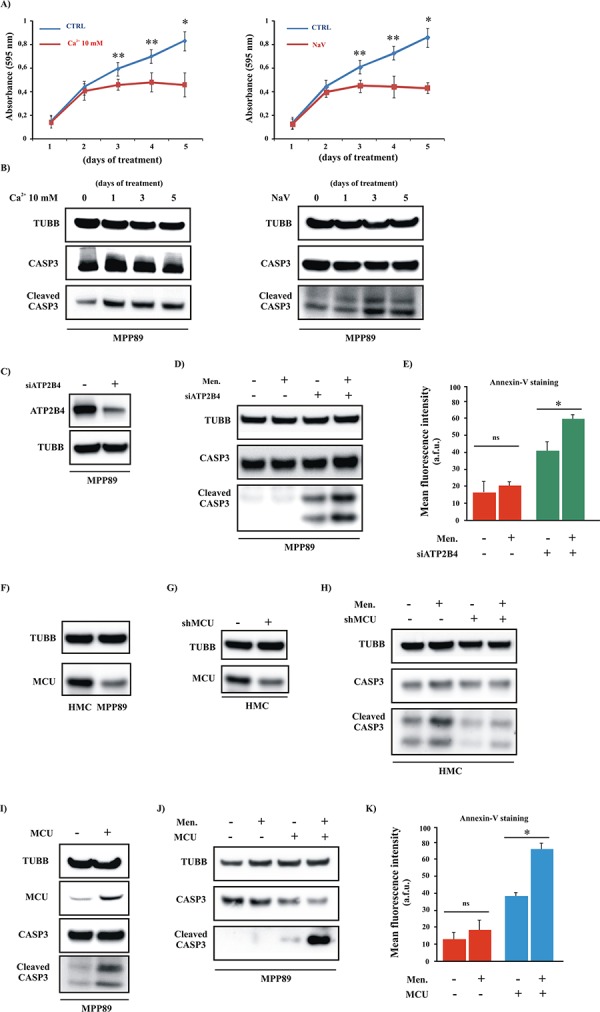
Positive modulation of Ca^2+^ handling restores the apoptotic machinery in MPM cell lines **A.** Growth curves of MPP89 cells after 1, 2, 3, 4 and 5 days of treatment with 10 mM Ca^2+^ (left) or NaV (right) ([CTRL]: 1 day: 0.14 ± 0.049, 2 day: 0.42 ± 0.069, 3 day: 0.57 ± 0.067, 4 day: 0.66 ± 0.056 and 5 day 0.80 ± 0.103; [Ca^2+^ 10 mM]: 1 day: 0.13 ± 0.072, 2 day: 0.39 ± 0.091, 3 day: 0.44 ± 0.037, 4 day: 0.46 ± 0.108 and 5 day 0.44 ± 0.112; [NaV]: 1 day: 0.13 ± 0.029 2 day: 0.38 ± 0.044, 3 day: 0.43 ± 0.039, 4 day: 0.42 ± 0.108 and 5 day 0.41 ± 0.031, *n* = 4). **B.** Mesothelioma cell lines were cultured in complete medium and treated with 10 mM Ca^2+^ (left) or NaV (right) for different periods (1, 2, 3, 4 and 5 days) and immunoblotted. **C–E.** Representative immunoblot (C–D) and Annexin-V assay (E) in siRNA-ATP2B4 (siATP2B4)-expressing cells. For both experiments, cells were transfected with siATP2B4. After 36 h, the cells were harvested and immunoblotted for the indicated proteins or loaded with Annexin-V. Where indicated, the cells were treated with 30 μM Men for 30 min. (Annexin-V fluorescence intensity: 15.78 ± 6.75 a.f.u [CTRL], 19.22 ± 3.26 a.f.u [Men], 41.27 ± 4.62 [siATP2B4], 59.45 ± 1.87 a.f.u. [siATP2B4+Men]). **F.** Representative immunoblot showing the protein expression level of MCU in normal and cancerous cells. **G–H.** HMC cells were transfected with shRNA-MCU (shMCU). After 36 h of transfection, the cells were lysed for immunoblot and primary antibodies for TUBB and CASP3 were used. Where indicated, the cells were treated with 30 μM Men for 30 min. **I–K.** Western blot and Annexin-V assays in MCU-expressing cells. For both experiments, cells were transfected with MCU plasmid. After 36 h, the cells were harvested and immunoblotted for the indicated proteins (I–J) or loaded with Annexin-V (K) Where indicated, the cells were treated with 30 μM Men for 30 min. (Annexin-V fluorescence intensity: 12.78 ± 3.89 a.f.u [CTRL], 16.27 ± 4.11 a.f.u. [Men], 37.92 ± 1.53 a.f.u. [MCU], 64.76 ± 2.34 a.f.u. [MCU+Men]). *p < 0.01, **p < 0.05.

After 5 days of incubation in [Ca^2+^] or NaV, the growth capacity of the cells was affected (Figure 5A): a crystal violet cell proliferation assay indicated that the mesothelioma cells were less proliferative in the presence of high external [Ca^2+^] or NaV. This reduction in cell number suggested that the sensitivity of these cells to apoptosis was restored. Indeed, after 5 days of pretreatment with either compound, the MPP89 cells exhibited reactivation of the apoptotic machinery (Figure 5B).

The inhibition of ATP2Bs promoted by NaV and the high external [Ca^2+^] should increase the [Ca^2+^]_i_ levels and, in turn, [Ca^2+^]_m_ uptake. Thus, we investigated the effect of NaV and 10 mM [Ca^2+^] on mitochondrial Ca^2+^ homeostasis. As illustrated in Figure 5B, a relevant increase in the cleavage of CASP3 was detected after just 3 days; this effect was maintained through the fifth day. Based on this finding, we measured the ability of the mitochondria to absorb Ca^2+^ after 3 days of treatment. Our hypotheses were confirmed: pretreated MPP89 cells exhibited significant augmentation of [Ca^2+^]_m_ uptake (Supplementary Figure S4A). In addition to this, we found that these compounds also considerably modulated the overall intracellular Ca^2+^ signaling (Supplementary Figure S4B–S4C).

Even if NaV is reported to be a potent inhibitor of ATP2B4, we cannot be sure that the observed effects are due to a selective and unique interference with ATP2B4 activity.

Thus, to rule out the possibility that we were observing spurious effects, we evaluated the impact of silencing ATP2B4 on mitochondrial Ca^2+^ uptake and the apoptotic process using RNA interference (RNAi). Silencing ATP2B4 in MPM cells expressing a mtAEQ restores the [Ca^2+^]_m_ uptake (Supplementary Figure S4A). In addition, the RNAi rescues the sensitivity of mesothelioma cells to apoptotic stimuli (Figure 5C–5E).

Because increasing the intracellular Ca^2+^ levels restored the ability of the mitochondria to take up Ca^2+^ and to initiate the apoptotic process in MPM cells, we examined whether the direct and positive modulation of [Ca^2+^]_m_ could rescue the sensitivity of mesothelioma cells to apoptotic stimuli.

Recent publications report the identification of the complex responsible for [Ca^2+^]_m_ uptake: the mitochondrial Ca^2+^ uniporter (MCU) complex [39]. From its molecular discovery, several studies have investigated the significance of mitochondrial Ca^2+^ uptake on specific cellular processes, including carcinogenesis, through the modulation of MCU expression levels [40]. For example, a recent study identified MCU down-regulation as a characteristic feature of some human colon and prostate cancer cells [40]. No studies have yet assessed MCU in the context of mesothelioma. Based on these data, we investigated the MCU protein levels in normal and mesothelioma cells. Interestingly, we found that MPM cells displayed a significant reduction in the basal MCU protein levels (Figure 5F). Next, we explored whether the modulation of MCU activity could modulate apoptotic cell death via regulation of [Ca^2+^]_m_ uptake in normal and MPM cells. HMCs were transfected using a specific shRNA to silence MCU expression. Compared to the shRNA control, cells transfected with shRNA-MCU (shMCU) showed a significant decrease in the MCU protein levels (Figure 5G) and a significant reduction of [Ca^2+^]_m_ in HMCs (Supplementary Figure S4D). Furthermore, silencing MCU induced a marked reduction in the apoptotic machinery and in the sensitivity to pro-apoptotic stimuli (Figure 5H).

Similar experiments were carried out in MPM cells. Contrary to HMCs, we investigated the effects induced by MCU overexpression in MPM samples.

First, we evaluated the ability of the MCU plasmid to modulate MCU expression. As expected, a marked increase in the MCU protein levels was found upon MCU overexpression (Figure 5I). Next, we tested the effectiveness of MCU in modulating [Ca^2+^]_m_. The data (Supplementary Figure S4A) demonstrated that MCU caused a marked enhancement of the increase in [Ca^2+^]_m_.

Then, we investigated the possible activation of the apoptotic cascade. Mesothelioma cells with forced increases in the expression of MCU protein displayed elevated apoptotic activity (Figure 5I). Finally, we explored whether the augmentation of MCU expression sensitized mesothelioma cells to apoptotic stimuli. As reported in Figure 5J–5K, we detected a prominent activation of the apoptotic machinery in MCU-expressing cells that were exposed to a Ca^2+^-dependent apoptotic stimulus.

### Increased [Ca^2+^]_m_ transport restores apoptosis in primary MPM cells

**Figure 6:**
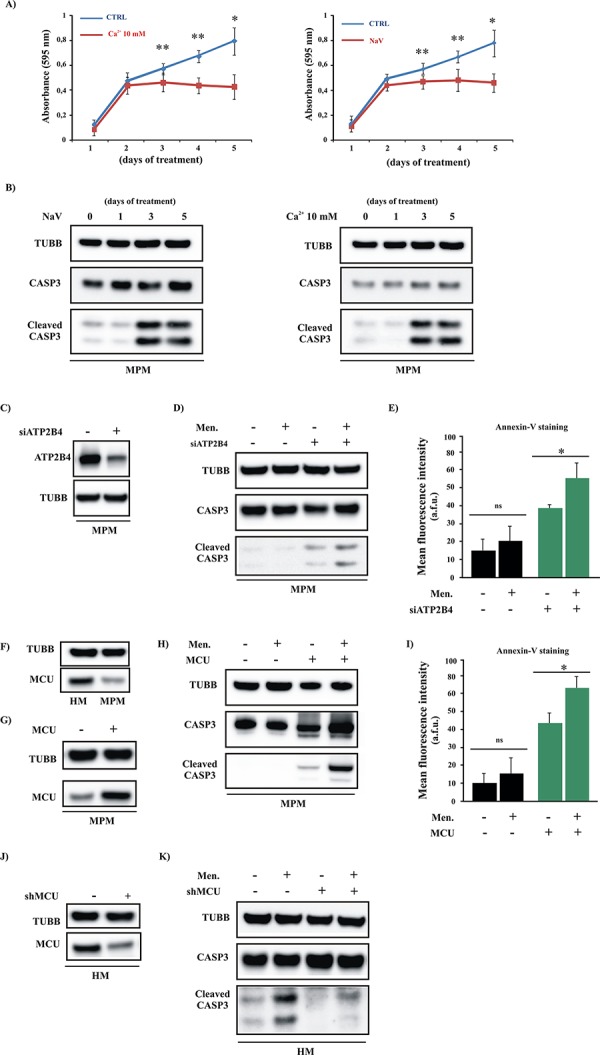
Increased mitochondrial Ca^2+^ transport restores the apoptotic process in primary MPM cells **A.** Primary cells obtained from MPM-affected patients were cultured in complete medium and exposed to a high external [Ca^2+^] (10 mM) or to NaV for different periods. After 1, 2, 3, 4 or 5 days of treatment, the cells were fixed for crystal violet cell proliferation assays ([CTRL]: 1 day 0.16 ± 0.021, 2 day: 0.53 ± 0.051, 3 day: 0.61 ± 0.029, 4 day: 0.70 ± 0.032 and 5 day 0.80 ± 0.126; [Ca^2+^ 10 mM, left panel]: 1 day 0.12 ± 0.023, 2 day: 0.45 ± 0.061, 3 day: 0.47 ± 0.058, 4 day: 0.45 ± 0.043 and 5 day 0.44 ± 0.115; [NaV, right panel]: 1 day 0.14 ± 0.038, 2 day: 0.45 ± 0.043, 3 day: 0.48 ± 0.058, 4 day: 0.49 ± 0.078 and 5 day 0.47 ± 0.068, *n* = 4). **B.** Primary cells obtained from MPM-affected patients (MPM) were cultured in complete medium and exposed to a high external [Ca^2+^] (10 mM) or to NaV for different periods. After 1, 2, 3, 4 or 5 days of treatment, the cells were lysed for immunoblotting. **C–D.** Representative immunoblot showing the cleavage of the apoptotic marker CASP3 in siRNA-ATP2B4 (siATP2B4)-expressing cells. **E.** Apoptotic measurements in siATP2B4-transfected MPM cells were also carried out by loading cells with Annexin-V. Where indicated cells were treated with Men for 30 min. (Annexin-V fluorescence intensity: 13.93 ± 4.98 a.f.u. [CTRL], 20.12 ± 8.92 a.f.u. [MEN], 39.05 ± 2.21 a.f.u. [siATP2B4], 55.01 ± 8.03 a.f.u. [siATP2B4 +Men] **F.** Immunoblotting showing the protein expression levels of MCU in HM and MPM cells. **G–I.** MPM cells were transfected with MCU plasmid and immunoblotted for the indicated proteins (G–H) or loaded with Annexin-V (I) to investigate the amount of PS on the outer leaflet of the PM (Annexin-V fluorescence intensity: 9.85 ± 4.63 a.f.u. [CTRL], 14.71 ± 5.38 a.f.u. [MEN], 42.75 ± 3.43 a.f.u. [MCU], 64.11 ± 4.73 a.f.u. [MCU+Men]). **J–K.** Downregulation of the MCU protein in HM cells after transfection with shRNA-MCU (shMCU) was confirmed by western blot analysis (K) Immunoblot showing the amount of cleaved CASP3 in total HM cell transfected with shMCU. TUBB was used as a loading control. Where indicated, the cells were transfected with shMCU (+) or control shRNA (−) and loaded with 30 μM Men for 30 min. *p < 0.01, **p < 0.05.

We attempted to reproduce this last set of experiments using primary cells obtained from biopsies of MPM-affected patients. MPM cells were pretreated with a high external [Ca^2+^] (10 mM) or with 1 μM NaV for 5 days, and the growth capacity of the cells was assessed. We found that similar to the mesothelioma cell lines, the primary mesothelioma short-term cultures were less proliferative in the presence of high external [Ca^2+^] or NaV after 5 days of incubation (Figure 6A). In parallel with the demonstrated involvement of the apoptotic machinery, the amount of cleaved CASP3 was increased (Figure 6B). Similar results were obtained after ATP2B4 silencing. In fact, compared to the siRNA control, cells transfected with siRNA-ATP2B4 (siATP2B4) showed a significant decrease in protein expression (Figure 6C), accompanied with a prominent increase in [Ca^2+^]_m_ (Supplementary Figure S4A). We also evaluated the effects of ATP2B4 silencing in modulating the apoptotic response. To assess whether ATP2B4 silencing modified the apoptotic process, we challenged siATP2B4-transfected MPM cells with the Ca^2+^-dependent apoptotic stimulus menadione. As result, the silencing of ATP2B4 promoted reactivation of the apoptotic machinery and sensitized mesothelioma cells to apoptotic stimuli (Figure 6D–6E).

Next, to examine the relationship between Ca^2+^ signaling and the apoptotic process, we evaluated whether direct modulation of [Ca^2+^]_m_ signaling was useful and necessary to enhance the sensitivity of cancerous cells to apoptosis-sensitizing cofactors. As first, and similarly to the mesothelioma cell lines, we found that cells obtained from biopsies of MPM-affected patients displayed lower amounts of MCU protein than HMCs (Figure 6F). This evidence confirms our hypothesis that MCU is a key player in mesothelioma disease. Thus, to increase the ability of mitochondria to absorb Ca^2+^, MPM cells were transfected with the MCU plasmid and treated with the Ca^2+^-dependent apoptotic stimulus menadione. The results obtained were very promising: the amount of active CASP3 and the fluorescence intensity of Annexin-V after the addition of menadione were significantly increased when the cells were transfected with MCU (Figure 6G–6I). Next, we aimed to confirm the importance of MCU during the Ca^2+^-dependent apoptotic process by promoting MCU-silencing in primary HMC samples. As expected, silencing of MCU reduces the ability of mitochondria to take up Ca^2+^ (Supplementary Figure S4D) and the MCU protein levels (Figure 6J). Of particular interest, we confirmed that shRNA-MCU (shMCU)-transfected HMC samples were less sensitive to apoptosis-sensitizing treatments than samples transfected with the control shRNA (Figure 6J–6K).

Overall these results highlight the role of MCU during MPM onset and demonstrate that by modulating Ca^2+^ handling, it could be possible to develop novel therapeutic approaches against this disease. Interestingly, treatments with NaV and high external [Ca^2+^] restore Ca^2+^ signaling and apoptosis without affecting MCU expression (Supplementary Figure S5A–5B), demonstrating the significance of correct ER-mitochondrial Ca^2+^ cross-talk as a primary contributor to the apoptotic program in mesothelioma.

## DISCUSSION

Few research studies have examined the importance of Ca^2+^ in MPM. Several investigations have indicated roles for different proteins in executing the mitochondrial (intrinsic) apoptotic pathway, and although many of these proteins are involved in Ca^2+^-handling, the direct relationship between Ca^2+^ and the regulation of apoptosis has yet to be addressed.

Among these proteins, BCL2 family members and the oncogene AKT were found to be dysregulated in mesothelioma cells [41–43]. Notably, it is widely appreciated that these proteins are also fundamental cofactors of the Ca^2+^-dependent pathway of apoptosis [9, 44]. Given these findings, our results associate the lack of apoptosis that is characteristic of mesothelioma cells with the core mechanism of the Ca^2+^-dependent apoptotic pathway. Notably, we found that MPM cells display a significant alteration in the expression levels of the apoptosis-related proteins reported above, including BCL2, BCL2L1 and AKT. Of relevance, in several tumor types the kinase AKT may exert its antiapoptotic function by regulating the type III ITPRs. Our results suggest that this feature is also preserved in mesothelioma disease. We found that the AKT-mediated ITPR3 phosphorylation was higher than in normal mesothelial cells. Furthermore, the levels of the oncosuppressor PML were reduced in MPM cells. PML was recently found to trigger the apoptotic program by modulating Ca^2+^ release from the ER [33]. This finding may support the hypothesis that MPM cells exhibit dysregulated Ca^2+^ signaling. The results reported in Figure 1 and Supplementary Figure S2 robustly support this model and demonstrate that the overall Ca^2+^ homeostasis of MPM cells is strongly affected.

Considering these results, we wondered whether Ca^2+^ is involved in the lack of apoptosis that is characteristic of mesothelioma cells. To address this question, we analyzed whether pro-apoptotic stimuli promote the apoptotic process via the employment of Ca^2+^ as an apoptosis-sensitizing cofactor. When normal mesothelial cells were exposed to apoptotic stimuli, we detected the progressive release of Ca^2+^ from intracellular stores, along with consequent alterations in mitochondrial energy and structure and, subsequently, the activation of caspases. In contrast, MPM cells were insensitive to these stimuli. Thus, these findings indicate that the observed reduction in Ca^2+^ homeostasis represents a pivotal mediator of the unresponsiveness of MPM cells to apoptotic stimuli. Studies are currently underway to elucidate the exact molecular mechanisms that underlie the downregulation of Ca^2+^ signaling which occurs in mesothelioma. Considering our results, we hypothesize that the PML-AKT-Ca^2+^ axis is directly involved in mesothelioma because a close relationship between AKT-PML and Ca^2+^-dependent apoptosis has already been demonstrated in other tumor types [33]. Furthermore, based on the identification of relevant alterations in Ca^2+^ flux across the PM and in the expression of specific Ca^2+^ channels and pumps, we also propose that these molecules actively participate in the pathogenesis of MPM. Consistent with this, silencing PMCA by RNA interference or NaV treatment restores the Ca^2+^ homeostasis and rescues the sensitivity of mesothelioma to apoptotic stimuli. Of relevance, the use of vanadate compounds as therapeutic agents has been reported [45, 46].

However, we do not exclude the role of other molecular pathways in the pathogenesis of MPM.

Indeed, the key role of BCL2/L1 in mesothelioma has been well demonstrated, and the use of pharmacological inhibitors of BCL2 family members is promising. Unfortunately, the development of these inhibitors requires further refinement [47]. Other studies have suggested the involvement of receptor tyrosine kinases (RTKs) in mesothelioma. Once activated, these kinases lead to the upregulation of two major downstream cell signaling cascades: the RAF1-MEK-RAS-ERK and PI3K-AKT-PTEN-MTOR pathways. Based on these findings, small molecule inhibitors of specific RTKs were applied in clinical studies, but they displayed only limited activity in advanced MPM patients [48, 49]. It is clear that several studies have identified molecules that mediate MPM, but a well-established molecular pathway and an effective molecular approach to combat this disease have yet to be discovered and developed.

Recent evidence has shown that specific cellular processes regulated by mitochondrial Ca^2+^ uptake, including carcinogenesis, are strictly supervised through the modulation and expression of the recent discovery channel (MCU) responsible for a correct [Ca^2+^]_m_ handling. For example, MCU silencing attenuates [Ca^2+^]_m_ [9, 10], and reduced [Ca^2+^]_m_ levels suppress cell death in a variety of cancerous cell lines, including those derived from the cervix, central nervous system and lymph tissues [44, 50, 51]. Furthermore, reduced MCU levels have been found in human samples of colon and prostate cells [40]. Nevertheless, the potential role for MCU to modulate cell death and other events in mesothelioma has not yet been addressed. Thus, having observed the critical dysregulation in overall Ca^2+^ handling and Ca^2+^-dependent apoptosis, we hypothesized that MCU could be affected in mesothelioma cells. The results reported in Figure 5F and 6F support this hypothesis and show that MPM samples display an overall reduction in MCU expression.

Based on these findings, we evaluated the involvement of the MCU in regulating the [Ca^2+^]_m_ levels and survival as a possible fundamental aspect of the pathology of MPM. The results were surprising. We detected a remarkable reduction in cell growth accompanied by an increase in the apoptotic response and a restored sensitivity to proapoptotic agents.

Interestingly, one of the most recent advances in cancer research is the demonstration that microRNAs (miRs) cause a variety of human disorders, particularly cancer [52, 53], and regulate the expression of genes encoding key regulatory molecules of Ca^2+^ signaling. Specifically, miR-25 was found to decrease mitochondrial Ca^2+^ uptake via specific MCU downregulation, conferring resistance to apoptotic stimuli [40]. One intriguing finding is that a comparative analysis of miR expression demonstrated that miR-25 was markedly upregulated in MPM cells [54]. Therefore, we sought to verify whether miR-25 may be a feature during the progression of MPM. Preliminary results (data not shown) suggest that inhibition of miR-25 may modulate apoptotic cell death via the enhancement of [Ca^2+^]_m_ uptake. In addition, a marked increase in the levels of MCU protein was found following miR-25 inhibition (data not shown).

Nevertheless it may be possible to consider miR-25 as a possible diagnostic marker and a novel therapeutic target, it is well known that a single miR can target multiple downstream transcripts and that many transcripts can be regulated by multiple miRs. Thus, further investigations are ongoing to discern the exact relationship between MPM, MCU and miR-25.

In conclusion, i) intracellular Ca^2+^ signaling plays an important role in MPM; ii) the downregulation of Ca^2+^ handling exerts an adverse effect on the execution of the apoptotic process and iii) on the sensitivity to apoptotic stimuli; iv) the recovery of Ca^2+^ homeostasis enhances Ca^2+^ uptake by the mitochondria, thereby promoting apoptotic activity; and v) MCU is a key mediator of MPM, and vi) its pharmacological modulation restores the rate of apoptosis and the sensitivity to apoptotic stimuli.

## MATERIALS AND METHODS

### Cell culture and transfection

Human normal mesothelial cells (HMs) were obtained from biopsies collected from young non-oncologic patients (age 29–35 ys) affected by pneumothorax at the Surgical Clinic of the University/Hospital, Ferrara.

MPM cell lines of commercial origin, such as MPP89, were from our cell line collections [54], and primary MPM cells were established from tumor biopsies obtained from patients affected by MPM [55]. HMCs were grown in RPMI medium 1640 (Euroclone), 10% FBS (Life Technologies), 2 mM L-Glutamine, and MPM cells were grown in D-MEM Ham's F12 (Euroclone), 10% FBS (Life Technologies). Cells were transfected using Lipofectamine 2000 or Lipofectamine LTX (Life Technologies) with the following plasmids: MCU and mtGFP. For aequorin measurements, cells were transfected with aequorin-targeted probes (mtAEQ, cytAEQ and erAEQ). For immunostaining, mitochondrial morphology analysis, ROS measurements cytochrome c staining and single-cell [Ca^2+^]_i_ measurements, cells were seeded on 24-mm glass coverslips. For aequorin measurements, the cells were seeded before transfection onto 13-mm glass coverslips and allowed to grow to 50–60% confluence. For the proliferation assay, cells were seeded on 12-well plates. For immunoblotting analysis, cells were seeded on 6-well plates with the same conditions for growth.

HMC and MPM cells were derived from surgical pleural samples of patients. Informed written consent was obtained from the patients to established MPM cell lines. This study was approved by the County Ethical Committee, Ferrara, Italy. Samples were obtained in sterile conditions and put in RPMI medium supplied with 50 U/ml of penicillin (CAMBREX) and 50 mg/ml of streptomycin (CAMBREX). The biopsy samples were cut in small pieces and transferred to 25-cm^2^ flasks containing RPMI 1640 medium supplemented with 10% FBS (CAMBREX), 2 mM L-glutamine (CAMBREX), 25 U/ml of penicillin (CAMBREX) and 25 mg/ml of streptomycin (CAMBREX) and added with 200U/ml of collagenase type 2 (Wortington). The samples were incubated at 37°C in a 5% CO_2_-humidified atmosphere over night to separate the cells from the tissue. The next day, the samples were washed with PBS 1X, mixed by pipetting, centrifuged for 10 minutes at 1000 r.p.m. and plated in new 25-cm^2^ flasks with 5 ml of complete medium. The cultures were maintained at 37°C in a 5% CO_2_-humidified atmosphere. The medium was changed twice a week, and the cultures were split according to their growth rate

Different morphological and proliferative parameters of HMC and MPM cultures were studied using immunofluorescence techniques. A panel of antibodies panel containing mouse anti-human MSLN HBME-1 (DAKO), goat anti-human CALB2 (SANTA CRUZ), mouse anti-human KRT 8–18 (SANTA CRUZ), and mouse anti-human VIM (SIGMA-ALDRICH) was used to characterized the cell lines. Immunofluorescence was carried out by covering fixed cells with 75 μl of the primary antibody diluted in PBS 1X as follows: mouse anti-human MSLN HBME-1 1:50, goat anti-human CALB2 1:50, mouse anti-human KRT 8-18 1:100, mouse anti-human VIM 1:200. The cells were incubated for 30 min at 37°C and subsequently washed three times in PBS 1X for 10 min. The slides were then dried on blotting paper and covered with 75 μl of secondary antibodies (anti-mouse or anti-goat) diluted in PBS 1X 1:50. The cells were incubated for another 30 min at 37°C and then washed three times in PBS 1X for 10 min. Finally, the samples were assembled on a chamber slide with 2 ml of glycerol-PBS 1X 7:3 supplemented with 0.5 mg/ml DAPI.

### Intracellular calcium measurements

The cytosolic Ca^2+^ response was evaluated using the fluorescent Ca^2+^ indicator Fura-2/AM (Life Technologies). Cells were grown on 24-mm coverslips and incubated at 37°C for 30 min in 1 mM Ca^2+^/KRB supplemented with 2.5 mM Fura-2/AM, 0.02% Pluronic F-68 (Sigma), 0.1 mM Sulfinpyrazone (Sigma). The cells were then washed and supplied with 1 mM Ca^2+^/KRB. To determine the Ca^2+^ cytosolic response, the cells were placed in an open Leyden chamber on a 37°C thermo-controlled stage and exposed to 340/380 wavelength light using the Olympus Xcellence multiple wavelength high-resolution fluorescence microscopy system equipped with a UAPON 40 × 0340-2 objective, numerical aperture 1.35 (Olympus). The fluorescence data were expressed as emission ratios. Fluorescence was measured every 100 ms, and the [Ca^2+^]_i_ was calculated by the ratio method using the equation: [Ca^2+^]_c_ 1/4 Kd (R–Rmin)/(R–Rmax) Sf2/Sf1.

To measure the thapsigargin-releasable Ca^2+^ levels, cells grown on 24-mm coverslips were incubated for 30 min in 1 mM Ca^2+^-free medium/KRB supplemented with 2.5 mM Fura-2/AM, 0.02% Pluronic F-68 (Sigma), 0.1 mM Sulfinpyrazone (Sigma) and EGTA 100 μM. The fluorescence was measured every 100 ms. After 2 min., thapsigargin 100 nM was added to the cells, and the amount of Ca^2+^ released was estimated by normalization of the 340/380 ratio.

### Aequorin measurements

All aequorin measurements were performed as previously described [56]. The cells were cultured on 12-mm glass coverslips and co-transfected at 70% confluence with 0.5 μg of aequorin (endoplasmic reticulum-targeted mutated aequorin, erAEQ, cytosolic aequorin, cytAEQ or mitochondria targeted aequorin, mtAEQ) and 1.25 μg of the indicated plasmid. Cells transfected with erAEQ were reconstituted with coelenterazine n (Tebu-Bio, Le-Perray-en-Yvelines, France), after ER Ca^2+^ depletion by incubation for 1 h at 4°C in KRB supplemented with 100 mM EGTA and 40 mM tBHQ (2,5-Di-tert-butylhydroquinone) (Sigma). The cells were then washed with KRB supplemented with 2% BSA and 1 mM EGTA. Cells that were transfected with cytAEQ and mtAEQ were reconstituted with coelenterazine (Synchem, Felsberg/Altenburg, Germany) for 2 h in KRB supplemented with 1 mM CaCl_2_. All aequorin measurements were carried out in 1 mM Ca^2+^/KRB (cytAEQ and mtAEQ) or 100 mM EGTA/KRB (erAEQ). ER refilling was trigger by perfusing with 1 mM Ca^2+^/KRB until a steady state was reached. An agonist was added to the same medium, as specified in the figures. The experiments were terminated by lysing the cells with 100 mM digitonin in a hypotonic Ca^2+^-rich solution (10 mM CaCl_2_ in H_2_O). The output of the discriminator was captured by a Thorn EMI photon-counting board and stored in an IBM-compatible computer for further analyses. The aequorin luminescence data were calibrated offline into [Ca^2+^] values using a computer algorithm based on the Ca^2+^ response curve of wild-type and mutant aequorins.

### Antibodies, reagents and immunoblotting

For immunoblot and immunostaining analysis, the following antibodies were used: anti-TUBB (SIGMA-ALDRICH, T8328), anti-GAPDH (Cell Signaling, 14C10), anti-MCU (SIGMA-ALDRICH, HPA016480), anti-CASP3 (Cell Signaling, 9665), anti-PARP1 (Cell Signaling, 9532), anti-TRPC-1 (Santa Cruz, sc20110), anti-TRPC-4 (Santa Cruz, sc15063), anti-ATP2Bs (Santa Cruz, sc201917), anti-HSPD1 (Santa Cruz, sc376240), anti-ATP2B4 (Abcam, ab2783), anti-PML (Santa Cruz, sc966), anti-AKT (Cell Signaling, 9272), anti-pTHR308 AKT (Cell Signaling, 13038), anti-BCL2 (Santa Cruz, sc7382), anti- BCL2L1 (Santa Cruz, sc8392), anti-Cytochrome C (Abcam, ab90529), anti-ITPR3 (BD, 610312), anti-pSER/THR AKT Substrate (Cell Signaling, 9611), anti-ATP2A2 (Cell Signaling, 4388), anti-ACTN (SIGMA-ALDRICH, A1978), anti-CANX (Santa Cruz, sc-11397). Other chemicals used are the following: Sodium orthovanadate (NaV, SIGMA-ALDRICH), menadione (SIGMA-ALDRICH), H_2_O_2_ (SIGMA-ALDRICH), C_2_-ceramide (C_2_, SIGMA-ALDRICH), thapsigargin (SIGMA-ALDRICH, T9033).

For immunoblotting, cells were scraped into ice-cold phosphate-buffered saline and lysed in a modified 10 mM Tris buffer pH 7.4 containing 150 mM NaCl, 1% Triton X-100, 10% glycerol, 10 mM EDTA and protease inhibitor cocktail. After 30 min of incubation on ice, the lysates were cleared via centrifugation at 12,000 g at 4°C for 10 min. Protein concentrations were determined using the Bio-Rad procedure. Protein extracts, 15 or 25 μg, were separated on 4–12% bis-tris acrylamide and 4–20% tris-glycine gels (Life technologies, NP0323 and EC6026) and electro-transferred to PVDF or nitrocellulose membrane according to standard procedures. Non-specific binding sites were saturated by incubating the membranes with TBS-Tween 20 (0.05%) supplemented with 5% nonfat powdered milk for 1 h. Next, the membranes were incubated overnight with primary antibodies, and the binding was assessed using appropriate HRP-labeled secondary antibodies [Life Technologies, A16104 (goat anti-rabbit) and A24512 (goat anti-mouse)] plus a chemiluminescent substrate (Thermo Scientific, 34080). The chemiluminescence signals were detected using ImageQuant LAS 4010 (GE Healthcare). The immunoblots shown are representative of at least four independent experiments.

### Immunoprecipitation

Immunoprecipitations were carried out using protein G-coated sepharose beads (GE Healthcare, Chalfont St. Giles, UK) following the manufacturer's instructions. For whole-cell extracts, cells were incubated on ice for 20 minutes in the following lysis buffer: 50 mM Tris-HCl (pH 7.4), 150 mM NaCl, 1% NP-40 and supplemented with protease and phosphatase inhibitors (2 mM Na_3_VO_4_, 2 mM NaF, 1 mM PMSF and protease inhibitor cocktail). The lysates were clarified by centrifugation for 10 minutes at 13200 rpm before use. The total protein content of the cell lysates was measured with the Lowry assay. The extracted proteins (1000 μg) were incubated overnight with an ITPR3 antibody at 4°C. The next day, beads were added to the lysates. Precipitation of the immune complexes was carried out for 3 h at 4°C. Afterwards, the beads were washed with PBS supplemented with phosphatase inhibitors and PMSF. The samples were proceed by SDS-PAGE and analyzed using a standard western blotting technique.

### Immunofluorescence

Images were taken with a Nikon Swept Field Confocal (Nikon). For each condition, at least 25 independent visual fields were counted. Mesothelioma cells were washed with PBS, fixed in 4% formaldehyde for 10 min and washed with PBS. Then, the cells were permeabilized for 10 min with 0.1% Triton X-100 in PBS and blocked in PBS containing 2% BSA and 0.05% Triton X-100 for 1 h. The cells were then incubated with primary antibody (anti-cytochrome c and anti-HSPD1) for 3 h at room temperature and washed three times with PBS. The appropriate isotype-matched, AlexaFluor-conjugated secondary antibodies [Life Technologies, A11008 (488 goat anti-rabbit) and A-21050 (633 goat anti-mouse)] were used. Images were obtained with a Nikon Swept Field confocal equipped with a CFI Plan Apo VC60XH objective (n.a. 1.4) (Nikon Instruments, Melville, NY) and an Andor DU885 EM-CCD camera (Andor Technology Ltd, Belfast, Northern Ireland). Statistical evaluation was performed using the colocalization counter JACOP, available in the Fiji software.

### RNA interference (RNAi)

The shRNA targeting MCU (TRCN0000133861) was purchased from Sigma-Aldrich. The siRNA targeting ATP2B4 (GS493) was purchased from Qiagen. shRNA and siRNA were transfected using the HiPerfect^®^ transfection reagent according to the manufacturer's instructions.

### Mitochondrial Ψ_m_

Mitochondrial Ψ_m_ was measured by loading cells with 20 nM tetramethyl rhodamine methyl ester (TMRM; Life Technologies) for 30 min at 37°C. The TMRM excitation was performed at 560 nm and emission was collected through a 590 to 650 nm band-pass filter. Images were taken every 5 s with a fixed 20-ms exposure time using a Nikon Swept Field confocal equipped with CFI Plan Apo VC60XH objective (n.a. 1.4) (Nikon Instruments, Melville, NY) and an Andor DU885 EM-CCD camera (Andor Technology Ltd, Belfast, Northern Ireland). FCCP (carbonyl cyanide p-trifluoromethoxyphenylhydrazone, 10 μM), an uncoupler of oxidative phosphorylation, was added after 12 acquisitions to completely eliminate the electrical gradient established by the respiratory chain. Where indicated, cells were pre-treated with menadione. The values are expressed in a.f.u. (arbitrary fluorescence units).

To establish the mitochondrial Ψ_m_ levels, the ratio between the residual TMRM fluorescence (obtained after FCCP addition) and the initial TMRM fluorescence was calculated. The values obtained represent the amount of the potential-indicating probe incorporated in the different experimental conditions.

### Mitochondrial morphology analysis

Mesothelioma cells were seeded onto 24-mm glass coverslips, allowed to grow to 50–60% confluence and then transfected with 1.5 μg of plasmid DNA mtGFP. After 36 h of expression, the cells were treated as described and then imaged with a Nikon Swept Field confocal microscope equipped with a CFI Plan Apo VC60XH objective (n.a. 1.4) (Nikon Instruments, Melville, NY) and an Andor DU885 EM-CCD camera (Andor Technology Ltd, Belfast, Northern Ireland). When indicated, the cells were treated with the reported compound. The coverslip were placed in an incubation chamber with controlled temperature, CO_2_ and humidity, and then 51-plane z-stacks were acquired with a voxel dimension of 133 × 133 × 200 nm (X Y × Z). The mitochondrial network was described in number and volume using the 3D object counter available in software Fiji (http://fiji.sc/wiki/index.php/Fiji, last accessed June 20, 2011).

### Detection of mitochondrial ROS

The cells were seeded onto 24-mm glass coverslips and allowed to grow to 60–70% confluence. The cells were then incubated with MitoSOX-Red indicator (Life Technologies Ltd) for 30 min and washed. When indicated, cells were treated with the pro-apoptotic compound menadione. After washing, the amount of mitochondrial superoxide was determined using a Tali™ image-based cytometer (Life Technologies). The data are presented as a histogram showing the % of the mean intensity of MitoSOX fluorescence.

### Growth curve assay

Cell growth curves were generated by crystal violet staining. Cells were seeded with a low cell density (5.000 per well) in 12-well plates in triplicate and allowed to grow for 5 days. At 1, 2, 3, 4 and 5 days, the cells were washed with phosphate-buffered saline, fixed with 4% paraformaldehyde and stained with 0.1% crystal violet. Crystal violet was dissolved with 1 mol/l acetic acid, and the A_595_ was measured.

### Statistical analysis

The results are expressed as the mean ± SEM, and the *n* refers to the number of independent experiments. The probability of significant differences between experimental groups was determined by Student's *t* test.

## SUPPLEMENTARY FIGURES


