# Supplementary material for: The endoplasmic reticulum mitochondrial calcium cross talk is downregulated in malignant pleural mesothelioma cells and plays a critical role in apoptosis inhibition
Source: Oncotarget. 2015 Jun 19;6(27):23427–44. doi: 10.18632/oncotarget.4370 (PMC4695128; doi:10.18632/oncotarget.4370)
Supplement: Supplementary file 1 [file oncotarget-06-23427-s001.pdf]

SUPPLEMENTARY FIGURES

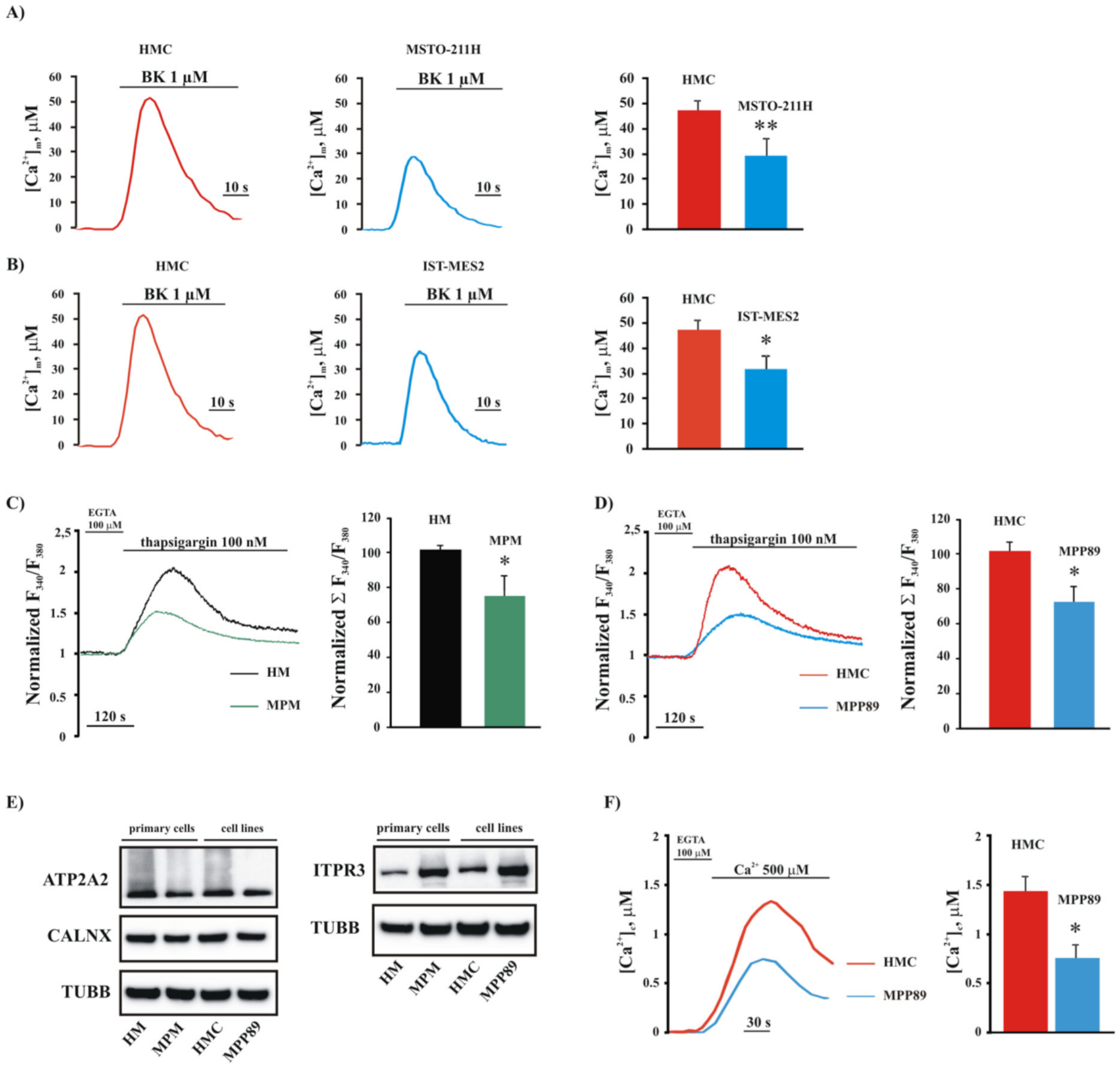

Supplementary Figure S1: Contribution of Ca^{2+} handling in MPM disease. **A–B.** Mitochondrial Ca^{2+} homeostasis measurements using aequorin in MSTO-211H (A) and IST-MES2 cells (B) The indicated cells were stimulated with BK. Representative traces of the $[Ca^{2+}]_m$ peaks are shown. The graphs display the means ± SEM. **C–D.** Thapsigargin-releasable Ca^{2+} in primary (C) and commercial (D) cells. Cells were loaded with the Ca^{2+}-indicator FURA-2/AM in KRB-Ca^{2+} free medium supplemented of 100 µM EGTA. After 30 min, cell were washed and imaged. Where indicated, cells were stimulated with 100 nM thapsigargin. The kinetic properties of the Ca^{2+} response are presented as the ratio of fluorescence at 340 nm/380 nm. **E.** Representative immunoblot showing the protein expression levels of the main $[Ca^{2+}]_{ER}$-regulator cofactors. TUBB was used as loading control. Experiments were carried out in normal and mesothelioma cell samples. **F.** Representative traces and graphs of cytosolic Ca^{2+}-uptake in normal mesothelial (HMC) and malignant mesothelioma (MPP89) cell lines. After Ca^{2+} depletion and aequorin reconstitution, the cells were transferred to a luminometer and perfused with 100 µM KRB/EGTA. Where indicated, the EGTA in the KRB was replaced with a final concentration of 500 µM $CaCl_2$. The graphs display the means ± SEM. *$p < 0.01$, **$p < 0.05$. Abbreviations: BK, bradykinin; KRB: Krebs ringer buffer.

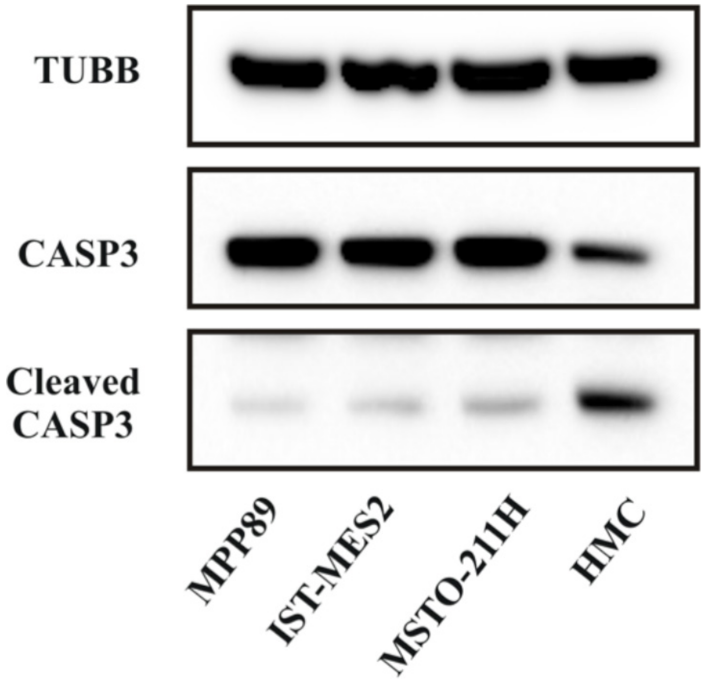

Supplementary Figure S2: Mesothelioma cells display reduced apoptotic activity. Mesothelioma cell lines (MPP89, IST-MES2 and MSTO-211H) were harvested and lysed, and the lysates were subjected to immunoblotting using an anti-CASP3 antibody. The same procedure was performed on the HMC cell line, and the lysate was used as a positive control for CASP3 cleavage. TUBB was used as a loading control.

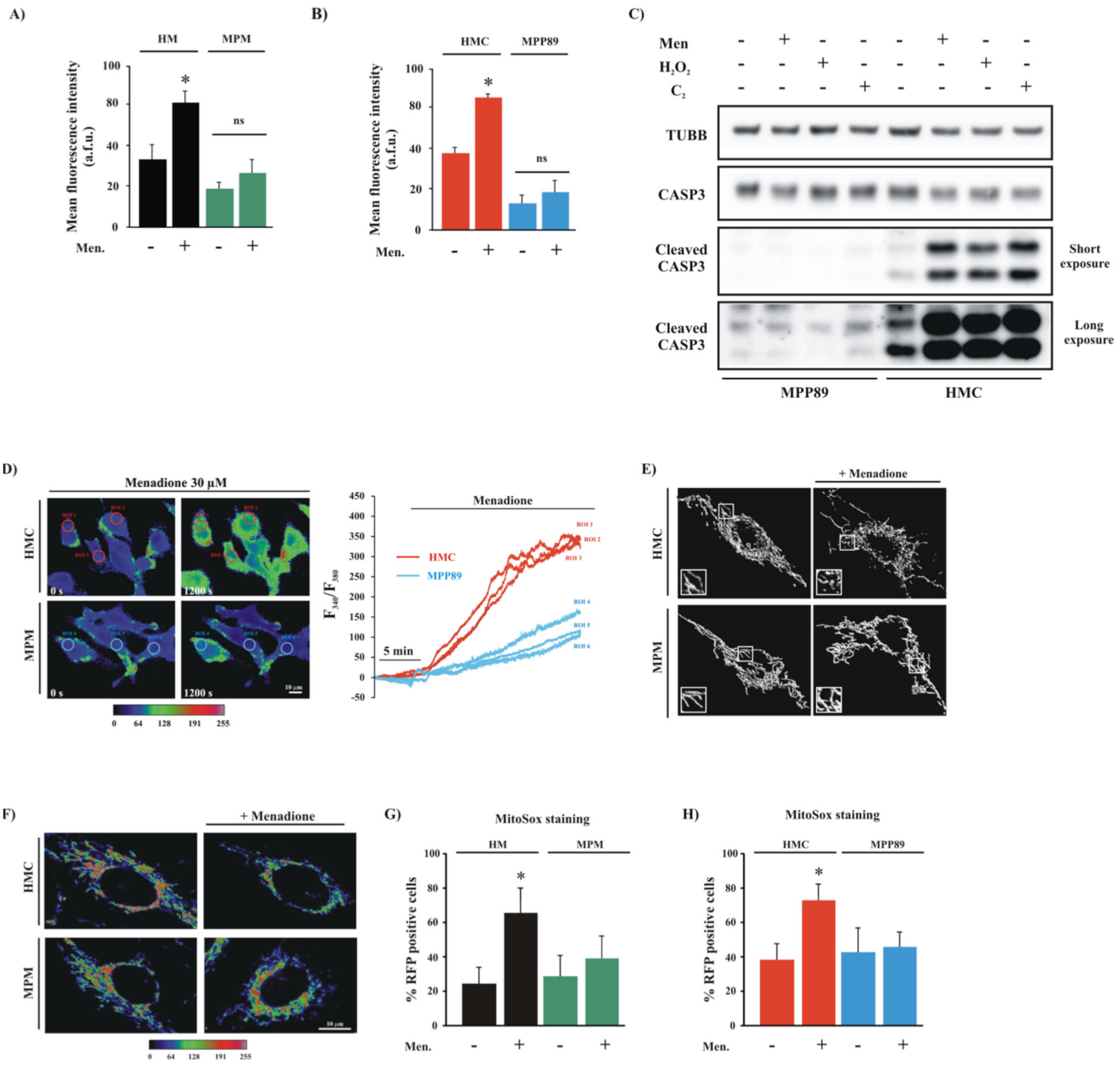

Supplementary Figure S3: ER-mitochondria Ca^{2+} dynamics are critically downregulated in mesothelioma cell samples. A. The fluorescence intensity of the Annexin-V-positive cells in the HMC and MPM cultures was detected using a TALI Image-Based Cytometer. Where indicated, cells were treated for 30 min with 30 µM Men. **B.** The same assay was reproduced in commercial normal (HMC) and mesothelioma (MPP89) cell lines **C.** HMC and mesothelioma cell lines were cultured in complete medium and then exposed to 100 µM H_2O_2 or 10 µM C_2-Ceramide (C_2) or 30 µM Men for 30 min. Next, the cells were harvested, and the lysates were subjected to immunoblotting using an anti- CASP3 antibody. TUBB was used as a loading control. **D.** Cytosolic Ca^{2+} response of HMC and MPP89 cells loaded with FURA-2AM dye upon menadione (30 µM) challenge. Microscopic fields of analyzed cells and the ratio of Fura-2 fluorescence 340 nm/380 nm averaged with the color-matched regions of interest (ROIs) are shown (red trace for HMC and blue trace for mesothelioma cells). Statistical analysis was reported in Figure 3D **E.** Representative 3D reconstituted confocal imaged of mitochondrial networks in normal and mesothelioma cell lines. Where indicated, cells were treated for 30 min with 30 µM Men. Statistical analysis was reported in Figure 3F. **F.** Representative images of HMC and MPP89 loaded with the potential sensitive dye TMRM. Cells shown are depicted on a pseudocolor scale with "warmer" colors on a rainbow scale corresponding to higher fluorescence. **G–H.** Analysis of mitochondrial superoxide production in HMC and MPM cells. After Men administration, the cells were loaded with the indicator MitoSox-Red for 30 min and washed three times. Next, the percentage of RFP-positive cells was assessed using a TALI Image-Based Cytometer. Experiments were carried out both in primary (G) and commercial (H) cell samples. *$p < 0.01$. Abbreviations: ns, not significant; a.f.u., arbitrary fluorescent units.

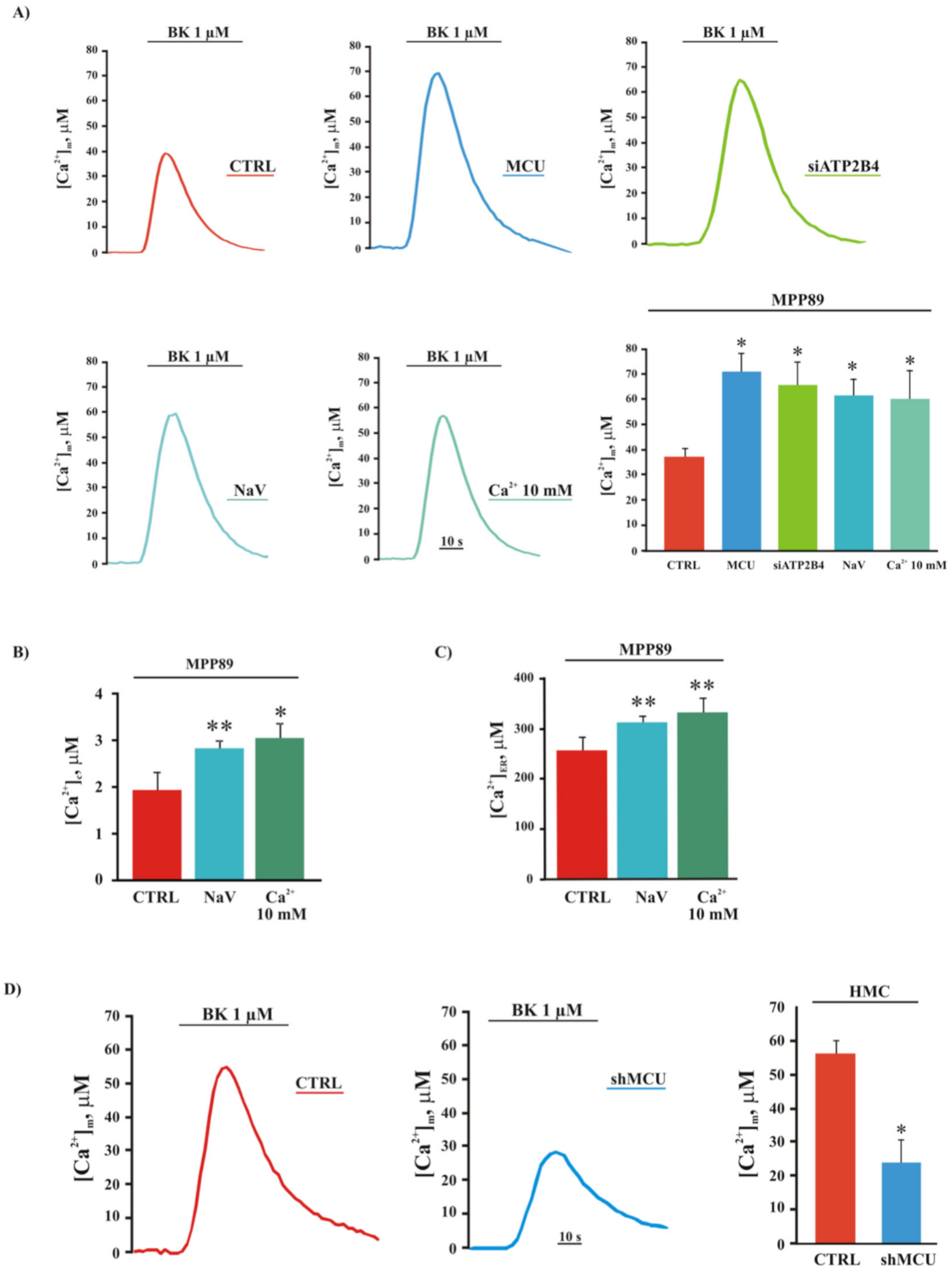

Supplementary Figure S4: Cofactors able to modulate Ca^{2+}-signaling amplify the mitochondrial Ca^{2+}-uptake in MPM cells. A. Mitochondrial Ca^{2+} measurements with mitochondrial targeted aequorin in MPM cell lines after treatment with high external $[Ca^{2+}]$ levels (Ca^{2+} 10 mM) or sodium orthovanadate (NaV) or transfection with MCU or siRNA-ATP2B4 (siATP2B4) plasmid. **B–C.** Graphs depicting intracellular Ca^{2+} dynamics ($[Ca^{2+}]_c$) values are shown in B; steady state $[Ca^{2+}]_{ER}$ levels are shown in C. Mesothelioma cells were treated with NaV or high external $[Ca^{2+}]$. **D.** The ability of mitochondria of HMC to adsorb Ca^{2+} was investigated following transfection with plasmid encoding shRNA-MCU (shMCU). Representative traces of $[Ca^{2+}]_m$ and quantitative results (mean ± SEM) are shown $*p < 0.01$, $**p < 0.05$. Abbreviations: BK, bradykinin.

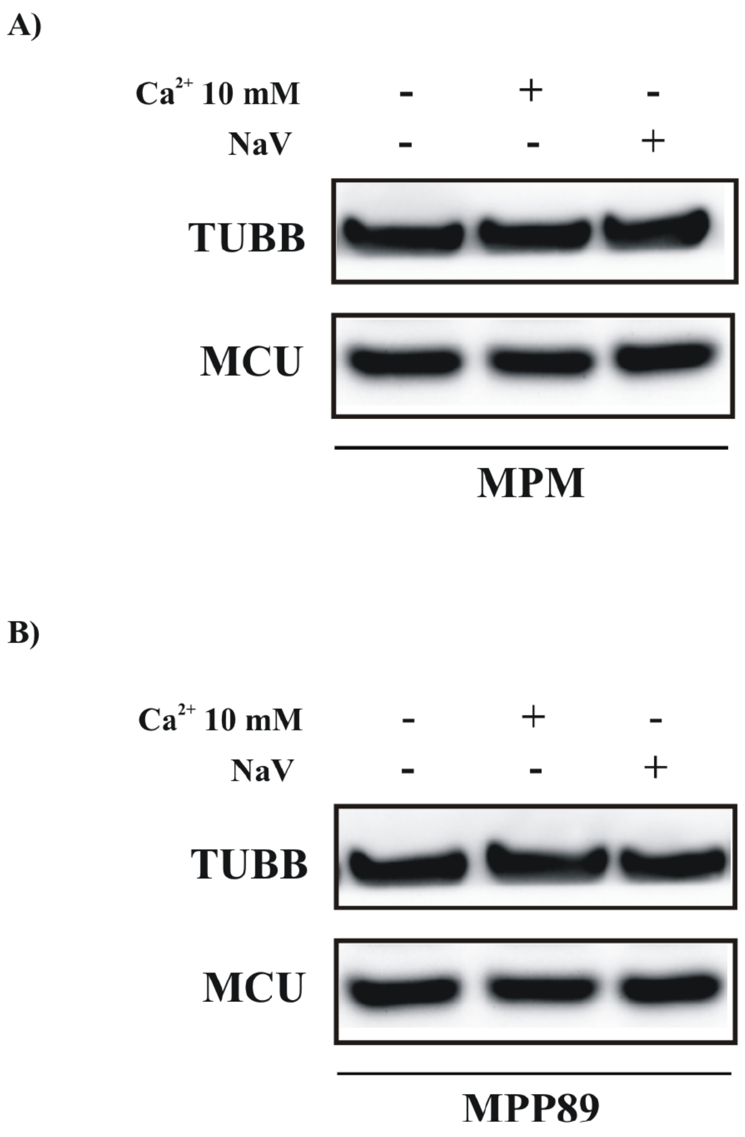

Supplementary Figure S5: Treatment with NaV and high external [Ca^{2+}] not modify MCU protein levels. Short-term cell cultures obtained from MPM-affected (MPM) patients and mesothelioma cell lines were cultured in complete medium and exposed to NaV and [Ca^{2+}] 10 mM. Next cells were collected and lysed for immunoblot analysis. The Western Blots shown are representative of three independent experiments.
